# A Systematic Review of Genetics- and Molecular-Pathway-Based Machine Learning Models for Neurological Disorder Diagnosis

**DOI:** 10.3390/ijms25126422

**Published:** 2024-06-11

**Authors:** Nasser Ali Aljarallah, Ashit Kumar Dutta, Abdul Rahaman Wahab Sait

**Affiliations:** 1 Department of Computer Science and Information Systems, College of Applied Sciences, AlMaarefa University, Ad Diriyah, Riyadh 13713, Saudi Arabia; adotta@um.edu.sa; 2 Department of Documents and Archive, Center of Documents and Administrative Communication, King Faisal University, Al-Ahsa, Al Hofuf 31982, Saudi Arabia

**Keywords:** genetics, molecular pathways, machine learning, speech disorders, neurogenetic disorder, neurodegenerative diseases, genomic data

## Abstract

The process of identification and management of neurological disorder conditions faces challenges, prompting the investigation of novel methods in order to improve diagnostic accuracy. In this study, we conducted a systematic literature review to identify the significance of genetics- and molecular-pathway-based machine learning (ML) models in treating neurological disorder conditions. According to the study’s objectives, search strategies were developed to extract the research studies using digital libraries. We followed rigorous study selection criteria. A total of 24 studies met the inclusion criteria and were included in the review. We classified the studies based on neurological disorders. The included studies highlighted multiple methodologies and exceptional results in treating neurological disorders. The study findings underscore the potential of the existing models, presenting personalized interventions based on the individual’s conditions. The findings offer better-performing approaches that handle genetics and molecular data to generate effective outcomes. Moreover, we discuss the future research directions and challenges, emphasizing the demand for generalizing existing models in real-world clinical settings. This study contributes to advancing knowledge in the field of diagnosis and management of neurological disorders.

## 1. Introduction

Neurological disorders cover a broader spectrum of conditions that affect the central nervous system, presenting significant challenges to healthcare professionals in diagnosis and disease management [1,2]. They may severely affect speech and language functions, resulting in various speech impairments [3]. The central nervous system is intricate, and numerous genes and their derivatives regulate neuron activity [4]. Genetics plays a significant role in neurological disorders, influencing their occurrence and progression [5]. Specific mutations in genes involved in brain, spinal cord, peripheral nerve, or muscle function lead to multiple neurological disorders [6]. For instance, Huntington’s disease or familial Alzheimer’s disease (AD) is caused by gene mutation. Neurogenetics has witnessed significant advancements, leading to a deeper understanding of the root causes of neurological disorders [7]. These breakthroughs have enabled whole-genome structure and variation analysis and human phenotypic analysis. Genome-based studies have highlighted the role of common genetic variability in influencing the risk for complicated disease development [8].

Genetics and molecular pathways are crucial to neurological disorder pathogenesis. Genetic variants, including single-nucleotide polymorphisms (SNPs), copy number variations (CNVs), and structural rearrangements, predict the likelihood of disease and progression in various neurological disorders [9]. Genome-wide association studies (GWAS) and next-generation sequencing (NGS) have revealed several genetic markers related to neurological conditions, revealing molecular pathways and enabling genetic testing and risk assessment [10]. Dysregulation of molecular pathways, including neurotransmitter signaling cascades, protein aggregation, immunological responses, and synaptic plasticity modes, contributes to neurological disorders [11,12]. Unique molecular pathways inside and across neurons influence neuronal function, maintenance, and survival, causing malfunction and disease. Molecular pathways are complex metabolic processes and signaling routes in neurons and glial cells of the nervous system [13]. These pathways are essential in maintaining protein homeostasis. The disruption of these pathways may cause developmental neurological disorders, including autism spectrum disorder or intellectual disability [14]. Neurological disorders are characterized by abnormal specific protein accumulation, for instance, amyloid-beta and tau proteins in AD and alpha-synuclein in Parkinson’s disease (PD) [15].

Traditional approaches have identified essential pathways associated with neuronal dysfunction, neuroinflammation, and synaptic impairment [16]. In recent years, ML has been ushered in a new era of data-driven discovery and innovation in biomedical research and clinical practice. ML algorithms can recognize complex patterns and can present valuable insights into neurological disorder diagnosis [17]. Researchers proposed several ML models to predict patient outcomes with exceptional accuracy and efficiency. The models, including support vector machine (SVM), decision trees, and random forest (RF), were used to identify biomarkers and patterns associated with neurological disorders. In particular, genetics- and molecular-pathway-based ML models have great potential to revolutionize diagnosis. The genetic architecture of disease susceptibility and progression may be revealed by analyzing whole-genome sequencing data to identify disease-related genetic variations [17]. ML algorithms can utilize transcriptomics, epigenomics, and proteomics data to understand neurological disorders complex molecular networks and pathways [18]. Recently, ML techniques have been integrated into DNA sequencing technology, allowing for the extraction of lengthy segments of DNA from digital electronic data [18]. Long-read techniques are crucial for resolving repetitive genomic areas and finding complicated structural variations [18]. These techniques can generate megabase-length DNA readings with optimal accuracy [18]. In addition, ML-based models have been used to classify genomic variation clinically by characterizing noncoding variants splicing code, DNA/RNA-binding proteins, and ncRNA utilizing massive molecular datasets [19].

The underlying mechanisms of neurological disorders may be better understood by using genetics and molecular data [20]. Researchers can employ these data to uncover the biological pathways and molecular signatures by analyzing genetic variants, protein interactions, and other molecular data. ML methods trained on these datasets can effectively differentiate healthy and unhealthy individuals, enabling early detection and interaction. Researchers can reveal unique relationships and patterns of neurological disorders by comparing genetics and molecular data obtained from multiple omics platforms [20]. Wekesa and Kimwele [21] presented the recent ML approaches for disease diagnosis using the omics data. They highlighted the recent trends in omics-data-based disease detection models. In addition, they provided data integration approaches for interpreting the model’s outcome. Bracher-Smith et al. [22] provided the psychiatric disorder detection models using a genetics dataset. They discussed the role of hyper-parameter optimization, validation methodologies, and predictor selections for psychiatric disorder detection. Ahmed et al. [23] described the datasets, feature extraction, and classification approaches for brain disorder detection. They analyzed SNP and genomic fingerprinting to track the disorder’s characteristics. However, these studies were limited to specific disorders and a lack of discussion related to diverse neurological and speech disorders. In addition, presenting the importance of data pre-processing, feature engineering, and classification techniques can assist the development of neurological and speech disorder detection models. In order to understand the potential of genetics- and molecular-pathway-based ML models, an extensive review of the current literature is essential. This systematic review intends to evaluate the reliability and clinical implementation of the ML models. In addition, it synthesizes the evidence from diverse studies to identify challenges and opportunities in leveraging genetics- and molecular-pathway-based ML models for improving neurological disorder diagnosis. The key contributions of this review are as follows:

1. The significance of genetics- and molecular-dataset-based ML approaches for neurological and speech disorder detection.
2. Presenting the features and limitations of genetics and molecular datasets.
3. Challenges and opportunities in developing the neurological and speech disorder detection models.

The remaining part of this review is structured as follows: Section 2 presents the proposed method for extracting research studies from the digital libraries. The review results are presented in Section 3. Section 4 offers the challenges and opportunities of the genetics- and molecular-dataset-based ML models. The review contributions and future directions are discussed in Section 5.

## 2. Methodology

In this section, we present the methodology for extracting and selecting the research articles. The research questions were formulated by identifying the knowledge gap in the existing literature. We conducted the review according to the PRISMA guidelines [24].

### 2.1. Research Questions

Research question 1: What are the ML techniques to identify neurological and speech disorders using genetics and molecular datasets?

Research question 2: What are the challenges in developing effective ML-based neurological and speech disorder detection models using genetics and molecular datasets?

### 2.2. Search Strategies

We developed a comprehensive search strategy to identify relevant literature from multiple resources, including Scopus, IEEE Xplore, PubMed, and the ACM digital library. The search strategy includes medical subject heading terms associated with neurological disorders. The details of the sources and keywords are outlined in Table 1. We combined search terms using Boolean operators, and we applied keywords using Boolean operators without time constraints. The subject area is used to extract a broader range of computer-science-related studies, including bioinformatics, health informatics, and other computer-aided neurological disorder detection.

### 2.3. Study Selection

We employed two independent reviewers to screen the titles and abstracts of search results in order to identify potentially relevant studies. The reviewers evaluated the full-text articles of the selected studies based on inclusion and exclusion criteria, as shown in Table 2. The discrepancies between the reviewers were addressed through consultation with a third reviewer.

**Figure 1:**
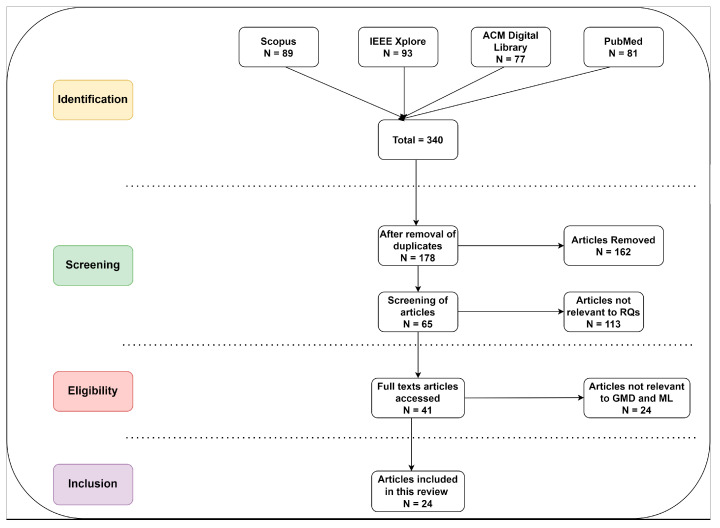
The flow of the extraction process.

Figure 1 highlights the extraction processes of the research studies. We focused on research questions and extracted articles about neurological and speech disorders. Initially, we extracted a total of 340 articles from digital libraries.

**Figure 2:**
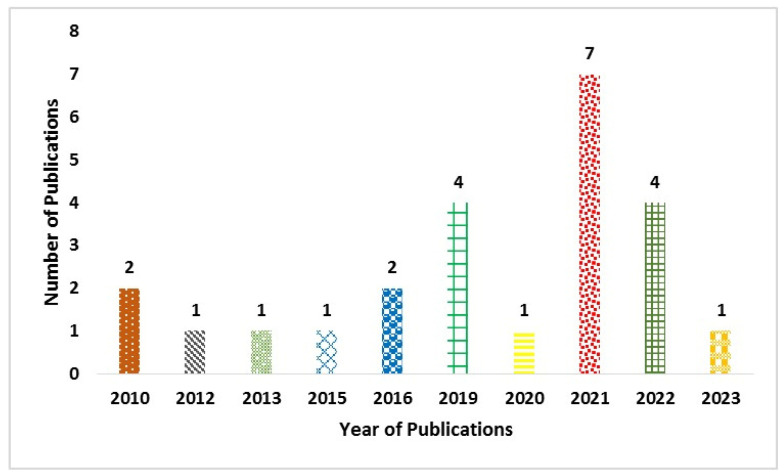
Year-wise publication of research studies.

A standardized data extraction form was used to extract data from the included studies. The data provide the author, publication year, study design, intervention details, and results. We used Microsoft Excel 2021 to evaluate the risks of bias and methodological quality. We exported the citations for each article in “.xlsx” format in order to evaluate them according to the selection strategies. Finally, we included a total of 24 articles. We extracted metadata, including year, title, methods, genetics and molecular datasets, type of classification/regression, evaluation metrics, and ground truth. Figure 2 shows the publication year of the research studies. It indicates the exponential growth of ML models based on genetics and molecular datasets in recent years. For instance, 17 articles were published between 2019 and 2023.

## 3. Results

We broadly classified the research articles into five types of neurological disorders, including AD, PD, autism spectrum disorder (ASD), schizophrenia (Scz), and other neurological disorders. We included research studies associated with multiple neurological disorders and presented an automated approach for predicting a rare neurological disorder or mental disorder. The majority of research studies employed standard evaluation metrics, including accuracy, the area under the receiver operating characteristics (AUROC) and precision–recall curve (AUPRC), sensitivity, and specificity. Data pre-processing, feature extraction using dimensionality reduction, and model development using neural networks (NN) were the fundamental techniques of the existing models. The recent developments in these techniques reduce computational time. However, few studies employed customized data pre-processing techniques to improve the model’s performance.

Most studies employed SNPs to extract the biomarkers associated with neurological disorders [25,26,27,28,29,30,31]. SNPs occur in genes, intergenic regions, and regulatory sequences, constituting the most straightforward genetic variation. The research studies utilized SNPs as input characteristics in genetics- and molecular-data-based ML models to predict disease risk, therapy response, and other clinical outcomes [32,33,34,35,36]. ML models were trained using SNP data and other factors to discover complex patterns and connections between genetic variants and phenotypic outcomes. Using SNP data, the existing models predicted disease risk, treatment response, and other clinical outcomes [37,38,39,40,41]. These models identify high-risk patients or guide personalized therapy.

### 3.1. AD Detection Models

Genetics- and molecular-pathway-based machine learning models can identify AD, uncover biomarkers, estimate risk, and customize therapy. These models can transform molecular findings into diagnostic and therapeutic techniques, extending fundamental research to clinical practice. In addition, these approaches promote evidence-based decision making and patient outcomes by integrating genetic and molecular data into clinical practice. The features of the research studies are presented in Table 3.

Huang et al. [25] analyzed the epigenetic data to identify kinases related to AD. An SVM classifier and RF classifier were used as base classifiers for kinase identification. The Beta Mixture Quantile Dilation algorithm was used to normalize DNA data. They performed microarray analysis to extract AD features. In addition, a feature selection technique was proposed to extract 5’—C—phosphate—G—3 (CPG) from DNA samples. Mirabnahrazam et al. [26] computed the dementia score for AD prediction using magnetic resonance imaging (MRI). Multimodal neuroimaging genomic data were used for AD prediction. The authors computed the dementia score for each individual using the brain volume and genetic features. An ensemble learning approach was followed to integrate the features in order to compute the final dementia score.

Alatrany et al. [27] proposed a strategy for AD prediction using genome data. Logistic regression was performed to identify the significant SNPs related to AD. They used the RF algorithm to identify the crucial features of AD. The Gini measure was used to assess feature importance. The authors used a genome-wide association studies (GWAS) dataset for the model evaluation. Using the multi-layer perceptron models, the model obtained an AUROC of 0.9 and 0.93. Monk et al. [28] proposed a NN model to investigate the impact of SNPs on AD. They employed exome data of ≈10,000 individuals. Data compression was used to transform raw data into a feature matrix. A multi-layer pattern recognition NN based on a feed-forward approach was developed for polygenic classification. The model identified the potential SNPs associated with AD with optimal accuracy.

### 3.2. PD Detection Models

Genetics- and molecular-pathway-based ML models guide PD genesis, revealing disease causes and treatment alternatives. Using genetic and molecular data, ML models may improve PD identification and management. The existing models identify high-risk PD patients based on genetic susceptibility and molecular markers. These models integrate genomic, transcriptomic, proteomic, and metabolomic data to improve diagnosis accuracy. Using insignificant disease-related patterns, they differentiate PD, atypical PD, and healthy controls compared to standard techniques. Table 4 highlights the importance of the PD detection models.

Bi et al. [29] proposed a multimodal approach using the MRI and genome data to predict PD. They constructed an evolutionary random NN for feature fusion analysis to integrate MRI and SNP data. They applied sample classification and PD association genes and brain region predictions. Pantaleo et al. [30] proposed a nested feature selection procedure using RF and XGBoost models. They used blood transcriptomic data for PD identification. A total of 493 candidate PD genes were extracted using the models. The nested feature selection procedure was followed to extract PD features. An RF classifier was used for feature importance analysis. Dadu et al. [31] proposed a model to predict PD subtypes and progression. Supervised and unsupervised learning models were used to analyze longitudinal clinical data. Genetic risk scores were computed for feature identification. Unsupervised clustering was used to determine the subtypes of PD. The experimental analysis was validated using the independent dataset. Ramezani et al. [32] examined the relationship between the SNCA gene and the cognitive abilities of PD individuals. They introduced a feature selection algorithm and integrated it with support vector regression to extract meaningful features. An SVM model was used to investigate the significance of gene variants on PD. The results revealed the rc894280 of the SNCA gene was associated with PD. Markarious et al. [33] presented a data analysis model using genome data to identify PD. They employed principal component analysis for feature extraction. ExtraTrees classifier was used to detect unique patterns of PD. They achieved an AUROC of 89.72.

### 3.3. ASD Detection Models

ASD is a complicated neurodevelopmental disorder with social communication difficulties and confined repetitive behaviors. The existing models promote early diagnosis, accuracy, and tailored medication by incorporating genetic variations and molecular pathways. These models improve ASD knowledge and clinical practice by discovering biomarkers, developing drugs, and conducting research. By revealing the intricate genetic, molecular, and environmental interactions that cause ASD, these models accelerate drug discovery and development. The characteristics of ASD detection models are listed in Table 5.

Zhan et al. [34] identified biomarkers associated with ASD, obsessive-compulsive disorder, and ADHD. They applied a stepwise logistic regression model to investigate the relationship between functional connections of a monkey-derived classifier and dimensional symptom severity of neurological disorders. They used the Least Absolute Shrinkage and Selection Operator (LASSO) to identify crucial ASD features. Statistical analysis was performed to detect abnormalities associated with ASD. The experimental evaluation was conducted using the functional connectivity data of methyl-CPG-binding protein in two transgenic monkeys and autism brain imaging data exchange. Ghatouri-Fard et al. [35] applied the artificial neural network (ANN) model for ASD prediction. The dataset was obtained from 487 ASD patients and 455 healthy individuals. A NN model was constructed to detect AD features. Scaled exponential linear units were used as an activation function to classify the features. A local interpretable modal-agnostic explanations model was used to interpret the model predictions. Engchuan et al. [36] proposed an ASD detection model using the Conditional Inference Forest technique. They extracted features using quality thresholds. The NN model was built using two middle layers to classify the extracted features. CNVs were used to classify ASD individuals. The model obtained a maximum AUROC of 0.533.

### 3.4. Scz Detection Models

Scz may cause hallucinations, delusions, chaotic thinking, and cognitive deficits. Using subtle disease-related patterns, ML models differentiate Scz from other psychological conditions better than traditional methods. They improve schizophrenia research by combining multiple data modalities and using cutting-edge algorithms.

Table 6 presents the characteristics of the ML models. Aguiar-Pulido et al. [37] developed a detection model for Scz using ML techniques. They used the naïve Bayes (NB) algorithm to identify the relationship among SNP genotypes. They trained SVM, NB, RF, and ANN for disease classification. SNPs were used to train the models. SVM model outperformed the other models by achieving an average accuracy of 94.2%. Likewise, Aguiar-Pulido et al. [38] used the ML techniques for Scz detection using genetic mutation. Quantitative genotype–disease relationships were used for disease detection. The features were extracted using NN and evolutionary computation models. A classifier based on disease relationships was used to detect Scz using the extracted features. Yang et al. [39] developed a fusion technique to integrate functional magnetic resonance imaging and genetic data for Scz detection. They used a feature selection AdaBoost and forward sequential feature selection methods to identify an optimal set of features. An SVM ensemble technique was developed to combine SNPs and functional MRI features. Vivian-Griffiths et al. [40] proposed a predictive modeling technique based on the SVM model to identify Scz. Using SNP imputation, linkage equilibrium, and Hardy–Weinberg equilibrium, they identified unique features of Scz. Polygenic risk score was used to predict the Scz risk. Trakadis et al. [41] employed a ML approach for Scz identification. They used LASSO regularized linear regression, RF, and extreme gradient boosting algorithms for feature selection. The performance evaluation was conducted using the Whole Exome Sequencing (WES) dataset.

### 3.5. Other Neurological Disorders

Pirooznia et al. [42] evaluated the performance of the ML approaches in identifying neurological disorders using genome data. They used a feature selection technique that performs clumping procedure to prune irrelevant SNPs. They computed polygenic scoring classifier for the disease classification. The experimental analysis was performed using the GWAS dataset. Guo et al. [43] applied ML approaches for detecting anorexia nervosa using genome genotyping data. They used LASSO-regularized logistic regression with a feature selection approach to find key patterns. Logistic regression with the LASSO penalty technique outperformed SVM and gradient-boosted trees by obtaining an AUROC of 0.693. Sardaar et al. [44] investigated the exome trios to identify the genomic architecture of neurological disorders. They followed a well-established population stratification correction method for feature selection. They used a clustering technique to identify significant genetic features.

Acikel et al. [45] built a genome classification model to find the association of SNPs with bipolar disorders. RF, naïve Bayes (NB), and K-nearest neighbor (KNN) were employed for the gene classification. The genotypes and phenotypes database (dbGap) dataset generalized the model. An open-source whole-genome association analysis toolkit was used to identify relationship between genotyping and phenotyping data. Bahado Singh et al. [46] performed an illumine Human Methylation 450K array in 23 cerebral palsy (CP) and 21 normal babies. They employed SVM and RF techniques to compute the methylation levels of genes. A genome-wide methylation analysis test was conducted to locate the crucial features. Partial-least-squares discriminative analysis was performed to detect CP. The findings highlighted the significance of AI techniques in predicting CP. The model provided mechanistic information on CP pathogenesis. Rita Singh [47] developed an AI-based algorithm using breadth-first analysis to find the significance of FOXP2 in voice disorder. A path-finding algorithm was used to determine the relationship among vocal characteristics and perturbing factors. A dataset of 42,784 gene symbols and 3245 gene families was used to validate the model. The results highlighted the crucial role of FOXP2 in voice production.

Magen et al. [48] identified microRNA (MiRNA) biomarkers of amptrophic lateral sclerosis (ALS) and frontotemporal dementia (FTD). They used the miRNeasy micro kit and Qubit flurometer to extract total RNA. A feature extraction technique based on ML approaches was used for biomarker extraction. The findings highlighted the exceptional performance of the model. Table 7 presents the characteristics of the neurological and speech disorders detection models.

### 3.6. Datasets

The large-scale GWAS dataset (https://www.ebi.ac.uk/gwas/, accessed on 15 November 2023) includes genetic and phenotypic data from several individuals. GWAS datasets are widely used to identify genetic variations associated with phenotypes. These connections may reveal the genetics of complex characteristics and disorders and provide treatment strategies. Meta-analyses, replication studies, and data sharing using GWAS datasets enable researchers to comprehend the genetic architecture of individual traits and diseases.

The Alzheimer’s Disease Neuroimaging Initiative (ADNI) dataset (https://adni.loni.usc.edu/, accessed on 15 November 2023) has significantly improved the understanding of Alzheimer’s and associated neurological disorders. It includes clinical, imaging, genomic, and other biological data from North American patients recruited at numerous locations. Their prediction models, biomarker identification, disease mechanism studies, and clinical trial effectiveness assessments have been extensively employed. ADNI databases are publicly accessible to researchers worldwide, boosting cooperation and accelerating AD research.

Biomarkers and clinical features associated with PD progression are being studied longitudinally in the Parkinson’s Progression Markers Initiative (PPMI) (https://www.ppmi-info.org/, accessed on 15 November 2023). The PPMI dataset has contributed to helping to comprehend PD by revealing disease progression, identifying biomarkers for early diagnosis and prognosis, and establishing novel therapies.

The dbGaP dataset (https://healthdata.gov/dataset/Database-of-Genotype-and-Phenotype-dbGaP-/th78-z3aq/data, accessed on 15 November 2023) is an open-source project maintained by the US National Institutes of Health. It stores and shares genotype and phenotypic data from human health and disease research investigations. Genetic information is in the form of sequencing or genotyping data collected from research subjects and is included in the dbGaP collection. These data may comprise human genome SNPs, insertions, deletions, and other genetic variations. Scientific advancement and knowledge of human genetics and complex disorders are accelerated through dbGaP’s data reuse, meta-analyses, and cross-study comparisons. A regulated access method, data usage constraints, and ethical and legal data sharing and protection rules apply to researchers requesting dbGaP data.

## 4. Discussions

We have addressed research questions by conducting a literature review on genetics- and molecular-pathways-based ML models. A total of 340 studies were identified in the initial stage. Finally, 24 studies were included in this review. The larger number of studies were published in 2021. The review findings highlighted the underpinning of the ML techniques in handling genetics and molecular data. The majority of ML models were used for feature extraction. In addition, SVM models were widely employed to identify the genes associated with neurological disorders. However, these models were used for binary classification. It is evident that SNPs play a crucial role in neurological disorder identification. The limited number of studies provided statistical and ground truth information on genetics and molecular data. Most studies generalized the ML model on a real-time dataset. Table 3, Table 4, Table 5, Table 6 and Table 7 highlight the datasets and performance of the models.

The review findings promote neurological disorder research and knowledge advancement. Researchers can utilize the findings to test current hypotheses, improve research topics, or develop novel ideas by analyzing the literature for gaps and inconsistencies. We evaluated the efficacy of the current ML-based neurological and speech disorder detection models using the existing scientific evidence. These findings may motivate clinicians and researchers to determine evidence-based practices and develop intervention studies.

The outcome of this review can support healthcare centers and researchers in implementing an effective ML model. Deploying genetics- and molecular-data-driven ML models in primary healthcare can assist physicians in identifying neurological disorders in the initial stages. Most neurological disorders are associated with speech disorders. Identification of neurological disorders in the early stages can prevent the adverse effects of speech disorders. In addition, multi-modal-based ML models enable healthcare centers to detect neurological disorders using invasive and non-invasive approaches.

### Challenges and Opportunities

Genome, transcriptome, proteome, and other omics data facilitate complicated biological operations. Maintaining high-quality data is the most challenging aspect of training ML models. There is a lack of accessibility to extensive amounts of valuable medical data. Medical professionals’ expertise is required to label samples to improve the data quality. Techniques including upsampling, Fourier transform, and augmentation should be employed to enhance the quality of image, audio, and text data to overcome the constraints associated with insufficient data.

Integrating diverse omics sources into an ML framework presents model development and interpretation challenges. Due to genetic and environmental disparities, models trained on one population or cohort may not generalize effectively. Understanding model predictions and biological processes is essential for developing ML-based healthcare applications. The black-box nature of the ML models may lead to a lack of interpretability. This limits the application of models established in one community or therapeutic environment to other settings or varied groups. Integrating ML models into healthcare processes can be challenging and resource intensive. Developing accessible ML models for neurological conditions can be complicated due to their diverse symptoms and development patterns. Data sources, including imaging technology, genetic sequencing methodologies, and clinical procedures, might vary, rendering datasets untrustworthy. Supervised learning ML model demands accurate data labeling. Clinicians’ subjective perceptions may lead to erroneous annotations in medical datasets. ML algorithms employ sensitive genetic and biochemical data, which may raise ethical and security issues. Genetic and molecular data integration poses privacy, consent, and security challenges. Patient confidentiality, informed permission, and data management are essential to protect research and clinical integrity.

GWAS and ADNI datasets were used to train the more significant number of ML models. Few studies followed the standard validation procedures [26,27,28,29,30,31,41,43]. There is a lack of training, validation, and information on testing procedures. The existing models were generalized using real-time data. However, there is limited ground truth information associated with data. Thus, the performance of these models may vary in a real-time environment. There is limited information related to hyper-parameter optimization to enhance the performance of the genetics and molecular data model. These limitations may reduce the implementation of the research studies in healthcare settings.

Integrating genetics, molecular pathways, and ML offers an effective platform for detecting and comprehending neurological disorders. The future direction of these combinations is to achieve significant breakthroughs in diagnosis, treatment, and personalized medicine. There will be a greater focus on developing interpretable models, enabling physicians to understand the model’s predictions. In addition, there will be a heightened focus on protecting individual’s privacy against unauthorized access to genetics and molecular data.

Future research may include genomics, transcriptomics, proteomics, and metabolomics data into genetics- and molecular-pathway-based machine learning models for neurological disorder diagnosis. These models can improve diagnostic accuracy and disease mechanisms by incorporating gene expression profiles and protein biomarkers. Genetics- and molecular-pathway-based machine learning models in clinical practice may demand specific technology, computing resources, and trained professionals. To achieve universal healthcare acceptance and accessibility, these models’ cost-effectiveness and scalability need to be thoroughly assessed. Despite their promise, genetics- and molecular pathway-based machine learning models require extensive clinical validation. Large-scale prospective studies, external validation cohorts, and longitudinal follow-up are essential to evaluate these models’ performance, dependability, and generalizability across varied patient groups and healthcare settings.

Time constraints may prevent the review from including the latest studies published after the search cutoff date. Recently published studies may have affected the review’s comprehensiveness. The identified neurological disorders, study populations, and study locations may limit the generalizability of the review findings. The included studies may vary in design, patient populations, data sources, and methods. It may be challenging to synthesize and draw conclusions due to heterogeneity.

## 5. Conclusions

This systematic review presented an overview of genetics- and molecular-pathway-based ML models. We identified diverse studies showcasing the ability to improve diagnostic accuracy and clinical decision making. The included studies demonstrated promising outcomes, underscoring the significance of ML models in the field of neurological disorder diagnosis and management. The findings revealed the importance of SNPs and the SVM model in identifying neurological and speech disorders. Most studies applied decision tree techniques for neurological disorder identification. Real-time datasets were used to generalize the models. However, we identified limitations such as small sample sizes, methodological heterogeneity, and the demand for substantial validation in real-world settings. Future research is warranted to address these limitations and uncover the capability of ML models. Developing interpretable and clinical action models and integrating multi-omics data should be considered in order to build an effective ML model. Furthermore, efforts should be directed to equitable access to advanced diagnostic technologies and accelerate the translation of research findings into clinical practice through interdisciplinary collaborations. The included studies may vary in design, participant characteristics, treatments, outcome measures, and other criteria. There has been a lot of recent progress in using ML models to identify neurological disorder based on genetics and molecular data. Systematic reviews necessitate continuous revisions to include new evidence and ideas.

## Figures and Tables

**Table 1:** Key terms and search string generation.

Sources	Terms
Scopus	Neurological disorder
IEEE Xplore	Speech disorder
PubMed	Molecular data
ACM digital library	Genetics
	Genome data
	Machine learning
	Neurodegenerative disease
(Neurological disorder) AND (Genetics OR Molecular OR Genome data) AND (Speech disorder) AND (Machine Learning) AND (LIMIT-To (Language, “English”)) AND (LIMIT-To (SUBJECT AREA, “computer science”))	

**Table 2:** Inclusion and exclusion criteria.

Inclusion	Exclusion
Studies presented in the English language Journal and conference articles Field of computer science No time constraints Related to genetics- and molecular-data-based neurological and speech disorders	Theories without practical implementation Review articles Research articles without details of the dataset

**Table 3:** Characteristics of AD detection models.

Authors	Methods	Data Type	Sample Size (Number of Individuals)	Extracted Features	Performance	Merits	Demerits
Huang et al. [25]	SVM and RF	DNA	717	334,465 autosamal CPG	AUROC = 0.962 AUPRC = 0.858	Feature ranking and adaptive hyperparameter search.	Findings may not be generalized to other neocortical regions.
Mirabnahrazam et al. [26]	Feature selection and ensemble learning	MRI and genetic	757	521,014 SNPs	Accuracy = 0.857	Sub-bagging approach-based training to overcome class imbalances.	Small sample size.
Alatrany et al. [27]	RF classifier	DNA	787	412,128 SNPs	AUROC = 0.90 using CNN and 0.93 using multi-layer perceptron	Multi-modality approach and feature variance reduction.	Trained in small dataset. Reducing feature set may lead loss of key data.
Monk et al. [28]	NN model	DNA	11,000	612,536 SNPs	Accuracy = 82.6%	Feature matrix transformation.	High computation cost.

**Table 4:** Characteristics of PD detection models.

Authors	Methods	Data Type	Sample Size (Number of Individuals)	Extracted Features	Performance	Merits	Demerits
Bi et al. [29]	Ensemble learning	MRI and DNA	90	23,595 SNPs	Accuracy = 88.57%	Multi-modality approach, feature fusion, and multi-task analysis.	Lack of generalization. The model may overfit the specific dataset.
Pantaleo et al. [30]	RF and XGBoost	Blood transcriptome	550	493 candidate genes	AUROC = 72%	Differential expression analysis-based biomarker extraction.	Specialized equipment and expertise are required for transcriptomic data analysis.
Dadu et al. [31]	Supervised and unsupervised learning	OMICS	440	64 PD bio-markers	AUROC = 0.92 CI = 95%	Biomarker-based prediction and multi-modality data analysis.	Lack of model interpretability.
Ramezai et al. [32]	SVM	MRI and blood transcriptome	101	11 PD bio-markers	Regression (*R*^2^) = 0.54	Multi-modality approach and hybrid feature selection technique.	Limited dataset and poor generalization.
Markarious et al. [33]	Genome-based data analysis model	Genetics	750	100 PD bio-markers	AUROC = 89.72	Low-cost model and ExtraTrees-based feature selection.	Lack of diversity in sample series.

**Table 5:** Characteristics of ASD detection models.

Authors	Methods	Data Type	Sample Size (Number of Individuals)	Extracted Features	Performance	Merits	Demerits
Zhan et al. [34]	Logistic regression	Blood transcriptome and MRI	336	94 ASD region of interest	Accuracy = 89.14% CI = 95%	LASSO-based feature selection and biomarker extraction based on core regions associated with ASD	The stepwise linear regression may not capture the non-linear relationships in neuroimaging data.
Ghafouri-Fard et al. [35]	ANN	DNA	942	15 SNPs	Accuracy = 93.67% AUROC = 80.59	Model interpretability using local interpretable model-agnostic explanations.	Risk of model overfitting in novel data and lack of longitudinal data.
Engchuan et al. [36]	Conditional Inference Forest	Genetics	4234	18,203 Copy number variation	AUROC = 0.533	Curated neurally relevant annotations-based predictive model and focus on rare CNVs.	Lack of generalizability and model performance is limited to CNVs detection technology.

**Table 6:** Characteristics of Scz detection models.

Authors	Methods	Data Type	Sample Size (Number of Individuals)	Extracted Features	Performance	Merits	Demerits
Aguiar-Pulido et al. [37]	SVM, NB, and Adaboost	DNA	614	48 SNPs	Accuracy: SVM = 94.2% NB = 90.4% Adaboost = 92.9%	Gene-specific analysis and linear NN-based classification.	Absence of external validation using independent datasets.
Aguiar-Pulido et al. [38]	SVM and NB	DNA	712	79 SNPs	Accuracy: SVM = 94.8% NB = 93.2%	Quantiative genotype and disease-relationship-based Scz classification.	Lack of model interpretability.
Yang et al. [39]	SVM	MRI and genetics	40	26 Scz bio-markers	Accuracy = 0.87	Multi-modality approach and feature fusion technique.	High computation costs and lack of generalization.
Vivian-Griffiths et al. [40]	SVM with non-linear and linear kernels	Genetics	11,853	4998 SNPs	AUROC = 0.662	Detection of non-linear genetic effects and interactions.	Non-linear SVM-based outcomes are complex and less interpretable.
Trakadi et al. [41]	Extreme gradient boosting with regularization	DNA	5090	112 SNPs	Accuracy = 85.7% AUROC = 0.95	Hybrid feature selection approach.	Limited scope of genetic variants.

**Table 7:** Characteristics of research studies based on other neurological and speech disorders.

Authors	Methods	Data Type	Sample Size (Number of Individuals)	Extracted Features	Performance	Merits	Demerits
Pirooznia et al. [42]	SVM, RF, RBF, and logistic regression	DNA	3625	1186 SNPs	AUROC: SVM = 0.515 RF = 0.521 RBF = 0.545	Polygenic score approach and extraction of potential genetic mechanisms.	The complexities of genetic effects may not be fully captured.
Guo et al. [43]	Logistic regression and SVM	Genetics	13,206	317,481 SNPs	AUROC = 0.693	LASSO-regualized logistic-regression-based classification.	The sensitiveness of LASSO regularization may lead to instability in the selected features.
Sardar et al. [44]	ML algorithm	Genetics	598	90 bio-markers	Accuracy = 86–88%	Focussing on rare variants and regularized-gradient-boosting-method-based disease classificaiton.	Limited dataset and absence of feature interpretation.
Acikel et al. [45]	RF, NB, and K-NN	Genetics	1767	1214 SNPs	Accuracy: RF = 0.734 NB = 0.702 K-NN = 0.733	Multi-factor dimensionality reduction and non-parametric model for SNP analysis.	Absence of model interpretability and high computational costs.
Bahado Singh et al. [46]	AI techniques	Blood transcriptome	44	230 differently-methylated CPG	AUROC ≥ 0.75 Sensitivity = 95.00% Specificity = 94.4%	Bio-informative and statistical anlaysis.	High computational costs and requirement of extenstive training.
Rita Singh [47]	Breadth-first-based path-finding algorithm	Genetics	4319	26 genes	Chainlink genes: Mean = 47.3 Median = 34.5 Chainlink connectivity: Mean = 704.3 Median = 514.7	Biomarker-extraction-based genomic data	The study outcomes are limited to a specific gene variant.
Magen et al. [48]	ML approaches	OMICS	219	132 miRNA predictors	ALS diagnosis: AUROC = 0.85 FTD diagnosis: AUROC = 0.70	miRNA biomarker extraction using a non-linear prediction model.	Lack of model interpretation.

## Data Availability

No new data were created or analyzed in this study. Data sharing is not applicable to this article.
